# Analytical modeling of uniaxial strain effects on the performance of double-gate graphene nanoribbon field-effect transistors

**DOI:** 10.1186/1556-276X-9-65

**Published:** 2014-02-08

**Authors:** George S Kliros

**Affiliations:** 1 Department of Aeronautical Sciences, Division of Electronics, Electric Power and Telecommunication Engineering, Hellenic Air-Force Academy, Dekeleia Attica GR-1010, Greece

**Keywords:** Graphene nanoribbons FETs, Uniaxial strain, Analytic ballistic model, Device performance metrics

## Abstract

The effects of uniaxial tensile strain on the ultimate performance of a dual-gated graphene nanoribbon field-effect transistor (GNR-FET) are studied using a fully analytical model based on effective mass approximation and semiclassical ballistic transport. The model incorporates the effects of edge bond relaxation and third nearest neighbor (3NN) interaction. To calculate the performance metrics of GNR-FETs, analytical expressions are used for the charge density, quantum capacitance, and drain current as functions of both gate and drain voltages. It is found that the current under a fixed bias can change several times with applied uniaxial strain and these changes are strongly related to strain-induced changes in both band gap and effective mass of the GNR. Intrinsic switching delay time, cutoff frequency, and *I*_on_/*I*_off_ ratio are also calculated for various uniaxial strain values. The results indicate that the variation in both cutoff frequency and *I*_on_/*I*_off_ ratio versus applied tensile strain inversely corresponds to that of the band gap and effective mass. Although a significant high frequency and switching performance can be achieved by uniaxial strain engineering, tradeoff issues should be carefully considered.

## Background

Graphene is a promising material for nanoelectronics due to its high carrier mobility at room temperature and excellent mechanical properties [1,2]. However, the on-current-to-off-current ratio of graphene channel field-effect transistors (FETs) is very small due to the lack of a band gap. As a result, monolayer graphene is not directly suitable for digital circuits but is very promising for analog, high-frequency applications [3]. A sizeable band gap can be created by patterning the graphene sheet into a nanoribbon using planar technologies such as electron beam lithography and etching [4,5]. The band gap of a GNR depends on its width and edge orientation. Zigzag-edged nanoribbons have a very small gap due to localized edge states. No such localized state appears in an armchair graphene nanoribbon (AGNR). Son et al. [6] have shown that the band gap of an armchair graphene nanoribbon (AGNR) arises from both the quantum confinement and the edge effects. In the presence of edge bond relaxation, all AGNRs are semiconducting with band gaps well separated into three different families *N*=3*p*, *N*=3*p*+1, and *N*=3*p*+2, with *p* an integer, and in each family, the gap decreases inversely to the ribbon width [6]. However, the band gap of the family *N*=3*p*+2 is significantly reduced, resulting in a close-to-metallic channel. This classification has proved very helpful in the study of AGNRs since investigating AGNRs of various widths an equivalent behavior of ribbons of the same family is revealed.

Strain has important effects on the electronic properties of materials and has been successfully employed in the semiconductor technology to improve the mobility of FETs [7]. For GNRs, it has been established that the band structure can be drastically modified by strain. As a result, it has been proposed that strain can be used to design various elements for all-graphene electronics [8]. The effect of strain on the electronic structure and transport properties of graphene sheets and its ribbons have been studied both theoretically [9-11] and experimentally [12-14]. Uniaxial strain can be applied by depositing a ribbon of graphene on transparent flexible polyethylene terephthalate (PET) and stretching the PET in one direction [12]. Moreover, local strain can be induced by placing the graphene sheet or ribbon on a substrate fabricated with patterns like trenches as it has been explored for achieving quantum Hall effect [15]. To date, however, no experimental works on applying uniaxial strain to narrow GNRs (of sub-10 nm width) have been reported.

In comparison to a graphene sheet, whose band gap remains unaffected even under large strains of about 20%, the band gap of GNRs is very sensitive to strain [16]. Since shear strain tends to reduce the band gap of AGNRs, most studies are concentrated to uniaxial strain. Uniaxial strain reduces the overlapping integral of C-C atoms and influences the interaction between electrons and nuclei. As a result, the energy band structure, especially the lowest conduction subbands and the highest valence subbands should be changed. Recently, the band structure and transport properties of strained GNRs have been theoretically explored using tight binding as well as density functional first-principles calculations [16-19]. It is found that uniaxial strain has little effect on the band structure of zigzag GNRs, while the energy gap of AGNRs is modified in a periodic way with a zigzag pattern and causes oscillatory transition between semiconducting and metallic states. Moreover, the band gaps of different GNR families show an opposite linear dependence on the strain which offers a way to distinguish the families. Tensile strain of more than 1% or compressive strain higher than 2% may be used to differentiate between the *N*=3*p*+1 and *N*=3*p*+2 families as their band gap versus strain relationship have opposite sign in these regions [18,20]. However, shear strain has little influence on the band structure of AGNRs. On the other hand, neither uniaxial strain nor shear strain can open a band gap in zigzag GNRs due to the existence of edge states [16].

Although several studies have investigated the band structure of strained AGNRs, only a few have been focused on the performance of strained GNR-FETs [21-24]. These studies are based on first-principles quantum transport calculations and non-equilibrium Green’s function techniques. It is shown that the I-V characteristics of GNR-FETs are strongly modified by uniaxial strain, and in some cases, under a 10% strain, the current can change as much as 400% to 500%. However, the variation in current with strain is sample specific [22]. On the other hand, although semi-analytical [25] or fully analytical models [26] for the I-V characteristics of unstrained GNRs-FETs have been proposed, no analytical model of GNRs-FETs under strain has been reported.

In this work, using a fully analytical model, we investigate the effects of uniaxial tensile strain on the I-V characteristics and the performance of double-gate GNR-FETs. Compared to top-gated GNR-FET, a dual-gated device has the advantage of better gate control and it is more favorable structure to overcome short channel effects [27]. Since significant performance improvement is expected for nanodevices in the quantum capacitance limit QCL [28], a double-gate AGNR-FET operating close to QCL is considered. High frequency and switching performance metrics of the device under study, as transcoductance, cutoff frequency, switching delay time, and power-delay time product are calculated and discussed.

## Methods

### Device model

#### Effective mass and band structure

**Figure 1:**
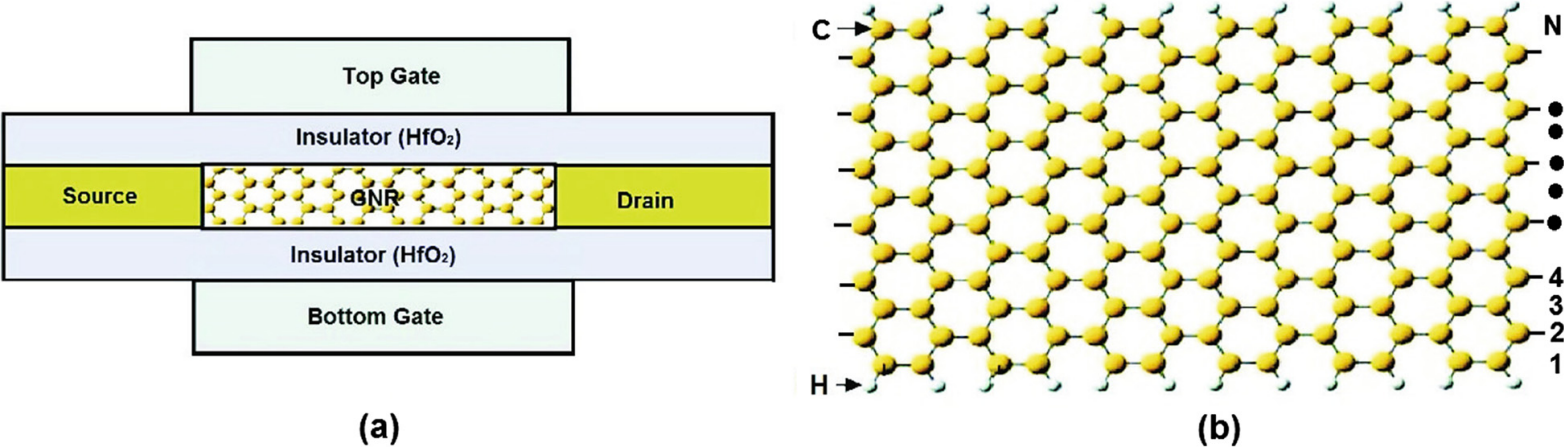
**Schematics of double-gate GNR-FET and the atomic structure of AGNR.(a)** Schematics of double-gate GNR FET where a semiconducting AGNR is used as channel material. **(b)** The atomic structure of AGNR. Hydrogen atoms are attached to the edge carbon atoms to terminate the dangling bonds. N is defined by counting the number of C-atoms forming a zigzag chain in the transverse direction.

The modeled GNR-FET has a double-gate structure with gate-insulator HfO_2_ of thickness *t*_ins_=1 nm and relative dielectric constant *κ*=16, as shown schematically in Figure 1a. The channel is taken to be intrinsic, and its length is supposed equal to the gate length *L*_G_. The source and drain contacts are heavily doped regions with doping concentration value of 5×10^−3^ dopants per carbon atom. Since thin and high- *κ* gate insulator is employed, we can expect excellent gate control to prevent source-drain direct tunneling. Moreover, the quantum capacitance limit (QCL), where the small quantum capacitance dominates the total gate capacitance, can be reached. The channel material is assumed to be a single-layer AGNR of the family *N*=3*p*+1, as it is illustrated in Figure 1b. It is well known that this family of AGNR is semiconducting material with promising characteristics for switching applications [26]. The edge boundaries are passivated by hydrogen atoms. It has been demonstrated that hydrogen passivation promotes the transformation of indirect band gaps to direct ones resulting in improved carrier mobility [19]. Moreover, the edge of the GNR is assumed to be perfect without edge roughness for assessing optimum device performance. In what follows, a power supply voltage of *V*_DD_=0.5 V and room temperature *T*=300 K are used.

Before dealing with the device performance under strain, we consider the effect of uniaxial strain on both band gap and effective mass of the AGNR. It has been verified that a 3NN tight binding model incorporating the edge bond relaxation can accurately predict the band structure of GNRs [29]. The 2NN interaction, which only shifts the dispersion relation in the energy axis but does not change the band structure, can be ignored. Any strain applied into the GNR modifies the C-C bonds accordingly. As a result, each hopping parameters in the tight-binding Hamiltonian matrix of the unstrained GNR is assumed to be scaled in Harrison’s form [30]*t*_*i*_=*t*_0_(*d*_*i*_/*d*_0_)^2^, where *d*_*i*_ and *d*_0_ are the C-C bond lengths with and without strain, respectively. Following the analysis of [16], where these changes are treated as small perturbations, we can express the energy dispersion of an AGNR under uniaxial strain in the form

(1) $$E_{n}^{\pm }(k_{x})=\pm \sqrt{E_{\text{C},n}^{2}+{\left(\hbar \upsilon_{n}k_{x}\right)}^{2}}$$

with

(2) $$\begin{array}{ll} E_{\text{C},n} & =c_{1}(\gamma_{1}+\gamma_{3})+2c_{2}\gamma_{1}s\;\text{cos}(\mathrm{n\theta})\\ & \;+2c_{2}\gamma_{3}\left[c_{3}+\left(1-c_{3}\right)\text{cos}(2\mathrm{n\theta})\right] \end{array}$$

and

(3) $${\left(\hbar \upsilon_{n}\right)}^{2}={\left(3a_{\text{cc}}\right)}^{2}\times \left\{\begin{array}{l} -\frac{1}{2}s\gamma_{1}c_{2}\text{cos}(\mathrm{n\theta})\left[c_{1}(\gamma_{1}+\gamma_{3})\right)\\ +\left(2c_{2}\gamma_{3}\left(c_{3}+\left(1-c_{3}\right)\text{cos}(2\mathrm{n\theta})\right)\right]\\ -\gamma_{3}\left[c_{1}\gamma_{1}+(c_{1}-1)\gamma_{3}\right)\\ +\left(2c_{2}\gamma_{3}\left(c_{3}+\left(1-c_{3}\right)\text{cos}(2\mathrm{n\theta})\right)\right], \end{array}\right)$$

where *θ*=*π*/(*N*+1), ± indicates the conduction band and valence band, respectively, *N* is the total number of C-atoms in the zigzag direction of the ribbon, *n* denotes the subband index, and *E*_C,*n*_ is the band edge energy of the *n*th subband. The strain parameters are expressed as *c*_1_=1+*α*, *c*_2_=1+*β*, *c*_3_=(*γ*_3_*c*_2_+*Δγ*_1_)/*γ*_3_*c*_2_(*N*+1) with *α*=−2*ε*+3*ε*^2^ and *β*=−(1−3*ν*)*ε*/2+(1−3*ν*)^2^*ε*^2^/4, where *ε* and *ν* are the strength of uniaxial strain and the Poissson ratio, respectively. Negative *ε* value corresponds to the compressive strain and positive *ε* value corresponds to the tensile strain. The first set of conduction and valence bands have band index *s*=−1. Due to the symmetric band structure of electrons and holes, one obtains for the energy gap *E*_G,*n*_=2*E*_C,*n*_. Also, *γ*_1_=−3.2*eV* and *γ*_3_=−0.3*eV* refer to the first- and third-nearest neighbor hopping parameters and *Δγ*_1_=−0.2 eV is used for the correction to *γ*_1_ due to edge bond relaxation effect. A poisson’s ratio value of 0.165 is used in this study [31]. The electron effective mass of each conduction subband can be calculated by using the formula

(4) $$m_{n}^{*}=\hbar^{2}{\left(\frac{\partial^{2}E_{n}(k_{x})}{\partial k_{x}^{2}}\right)}^{-1}$$

and at the bottom of the conduction band is given by

(5) $$m_{n}^{*}=\frac{E_{\text{C},n}}{\upsilon_{n}^{2}}.$$

**Figure 2:**
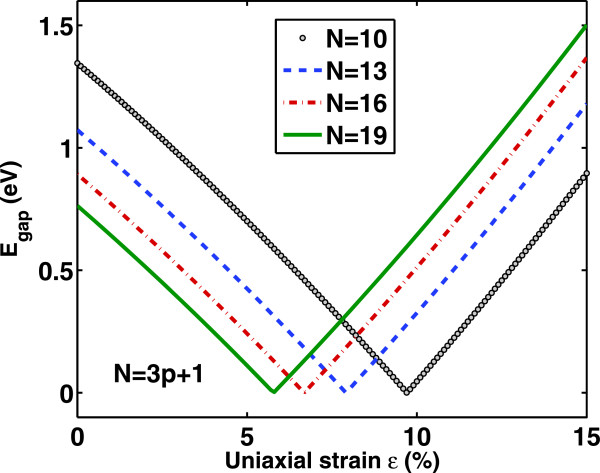
**Band gap variation versus uniaxial tensile strain for different (3** ***p*** **+1)-GNRs with indices** ***p*** **=3,4,5,6.**

**Figure 3:**
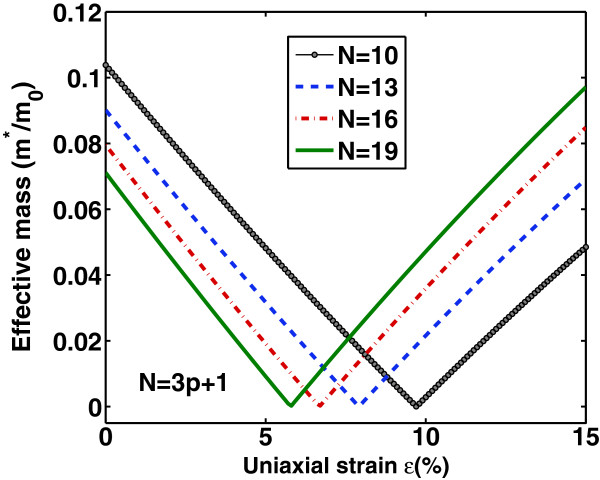
**Effective mass variation versus uniaxial tensile strain for different (3** ***p*** **+1)-GNRs with indices** ***p*** **=3,4,5,6.**

Figure 2 illustrates the dependence of band gap *E*_G,*n*_ of the GNR’s family *N*=3*p*+1 on the uniaxial tensile strain *ε*. As it is seen, in the range of tensile strain 0*%*≤*ε*≤15*%*, *E*_*g*_ decreases first and then increases linearly. Therefore, there is a turning point, i.e., as the strain increases, there is an abrupt reversal in the sign of *dE*_*g*_/*dε*, making the curves to display a V shape. The turning point moves toward smaller values of strain as the width of the AGNR increases. Moreover, the slope of *E*_*g*_(*ε*) is almost identical for various N and the peak value decreases with increasing N. The above observations are in agrement with the main features revealed by using tight-binding or first-principles numerical calculations [17,20]. On the other hand, Figure 3 shows the variation of effective mass at the conduction band minimum with strain *ε*. As it is clearly seen, $m_{n}^{*}$ has similar strain dependence as *E*_*g*_ and a linear relation between $m_{n}^{*}$ and *E*_*g*_ is expected which could be correlated to an inverse relationship between mobility and band gap [32]. These effective mass variations is attributed to the change in the conduction band minimum position under various strain values.

#### Device performance

Assuming a ballistic channel, the carriers with +*k* and −*k* states are in equilibrium with Fermi energies of the source (*E*_FS_) and the drain (*E*_FD_), respectively, with *E*_FS_=*E*_F_ and *E*_FD_=*E*_F_−*qV*_D_. The carrier density inside the channel can be obtained by employing the effective-mass approximation and integrating the density of states over all possible energies [26]

(6) $$n_{\text{1D}}=\sqrt{\frac{k_{B}T}{2\pi \hbar^{2}}}\underset{n>0}{\sum }\sqrt{m_{n}^{*}}\left[F_{-1/2}(\eta_{n,S})+F_{-1/2}(\eta_{n,D})\right],$$

where *F*_*j*_ is the Fermi-Dirac integral of order *j* defined as

(7) $$F_{j}(\eta )=\overset{\infty }{\underset{0}{\int }}\frac{x^{\;j}}{1+\text{exp}[\;x-(\eta /k_{\text{B}}T)]}\text{dx}$$

and *η*_*n*,*S*_=(*E*_FS_−*E*_C,*n*_)/*k*_B_*T*, *η*_*n*,D_=(*E*_FD_−*E*_C,*n*_)/*k*_B_*T*.

Considering the electrostatics describing the structure, the following relation between the gate voltage and Fermi energy *E*_F_ can be obtained [33]

(8) $$V_{\text{G}}(E_{\text{F}})-V_{\text{FB}}=\frac{E_{\text{F}}}{q}+\frac{{\text{qn}}_{\text{1D}}(E_{\text{F}})}{C_{\text{ins}}},$$

where *q* is the carrier charge, *C*_ins_ is the gate-insulator capacitance per unit length of the GNR and *V*_FB_ denotes the flat-band voltage. The value of *V*_FB_ depends on the work function difference between the metal-gate electrode and the GNR, and it can be set simply to zero as it is discussed in detail in [34]. The gate-insulator capacitance can be calculated by the simple expression [35]

(9) $$C_{\text{ins}}=N_{\text{G}}\kappa \varepsilon_{0}\left(\frac{W}{t_{\text{ins}}}+\alpha \right),$$

where *N*_G_ is the number of gates (*N*_G_=2 in our DG-device), *κ* is the relative dielectric constant of the gate insulator, *t*_ins_ is the gate-insulator thickness and *α* is a dimensionless fitting parameter due to the electrostatic edge effect. In our numerical calculation, a value of *α*=1 is adopted following [35]. The gate insulator capacitance increases linearly as the GNR width increases because the area of the GNR increases proportionally.

The bias-dependent gate capacitance per unit length *C*_*g*_ can be modeled as a series combination of insulator capacitance per unit length *C*_ins_ and the quantum capacitance per unit length *C*_Q_, that is,

(10) $$C_{g}=\frac{C_{\text{ins}}C_{\text{Q}}}{C_{\text{ins}}+C_{\text{Q}}}.$$

The quantum capacitance describes the change in channel charge due to a given change in gate voltage and can be calculated by *C*_Q_=*q*^2^*∂n*_1D_/*∂E*_F_ where *q* is the electron charge and *n*_1D_ is the one-dimensional electron density [33]. Using Equation (6) and writing in terms of Fermi integrals of order (−3/2), we obtain [26]

(11) $$C_{\text{Q}}=\frac{q^{2}}{{\left(2\pi \hbar^{2}k_{\text{B}}T\right)}^{1/2}}\;\underset{n>0}{\sum }\;\sqrt{m_{n}^{*}}\left[F_{-3/2}(\eta_{n,S})\;+\;F_{-3/2}(\eta_{n,\text{D}})\right].$$

Following Landauer’s formula and Natori’s ballistic theory [34,36], the device current is expressed by a product of the carrier flux injected to the channel and the transmission coefficient which is assumed to be unity at energies allowed for propagation along the channel. Contribution from the evanescent modes is neglected. Thus,

(12) $$I_{\text{D}}=\frac{q}{\pi \hbar }\underset{n>0}{\sum }\left[\overset{\infty }{\underset{E_{\text{C},n}}{\int }}\left(\;f_{\text{S}}(E)-f_{\text{D}}(E)\right)\text{dE}\right],$$

where *f*_S,D_(*E*) are the Fermi-Dirac probabilities defined as

(13) $$f_{\text{S,D}}(E)=\frac{1}{1+e^{(E-E_{\text{FS,D}})/k_{\text{B}}T}}.$$

After integrating, Equation (12) yields

(14) $$I_{\text{D}}=\frac{qk_{\text{B}}T}{\pi \hbar }\underset{n>0}{\sum }\ln\left[\frac{1+\text{exp}(\eta_{n,\text{S}})}{1+\text{exp}(\eta_{n,\text{D}})}\right].$$

For a well-designed DG-FET, we can assume that *C*_ins_≫*C*_D_ and *C*_ins_≫*C*_S_ which corresponds to perfect gate electrostatic control over the channel [28]. Moreover, carrier scattering by ion-impurities and electron-hole puddle effect [37] are not considered, assuming that such effects can be overcome by processing advancements in the future. In what follows, a representative AGNR with *N*=16 is considered.

## Results and discussion

**Figure 4:**
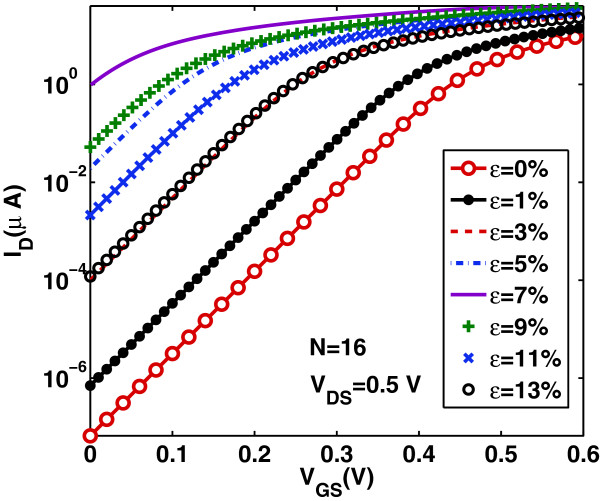
**Transfer characteristics** ***I***_**D**_**−*V***_**GS**_** for various tensile strain values.**

**Figure 5:**
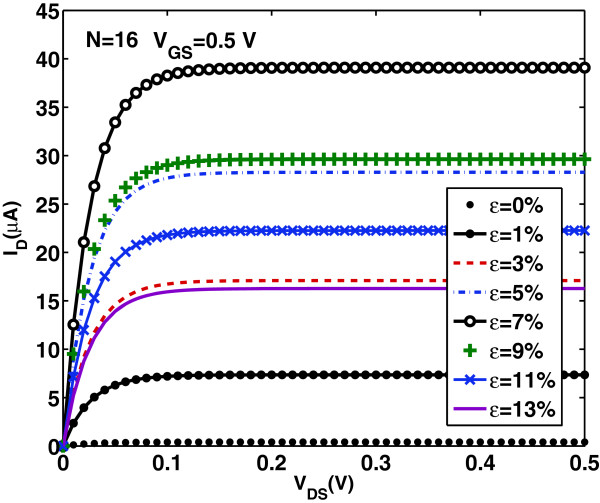
**Output characteristics** ***I***_**D**_**−*V***_**DS**_** for various tensile strain values.**

In this section, we firstly explore the calculated device characteristics. Figures 4 and 5 show the transfer *I*_D_−*V*_GS_ and output *I*_D_−*V*_DS_ characteristics, respectively, in the ballistic regime, for the DG AGNR-FET of Figure 1 with *N*=16, which belongs in the family *N*=3*p*+1, for several increasing values of uniaxial tensile strain from 1% to 13%. The feasibility of the adopted range of tensile strain values can be verified by referring to a previous first-principles study [22,23]. As it is seen from the plots, the current first increases for strain values before the turning point *ε*≃7*%* in the band gap variation (see Figure 2) and then starts to decrease for strain values after the turning point. Moreover, the characteristics for *ε*=5*%* are very close to that of *ε*=9*%*, and the same can be observed when comparing the characteristics of *ε*=3*%* with that of *ε*=13*%*. Note that, in each region of strain values (region before the turning point and region after the turning point), there is an inverse relationship between the current and the band gap values. Similar features in the current-voltage characteristics have been observed in the numerical modeling of [22,23] under uniaxial strain in the range 0≤*ε*≤11*%*. These features could be explained by the inverse relationship between mobility and energy gap which results to an increase in carrier’s velocity before the turning point and to the reduction in carrier’s velocity after the turning point [32]. It is also worth noting that, at the ballistic transport limit without electrostatic short channel effects, the characteristics in Figure 5 are independent on the channel length. This result is different from conventional FETs and can be explained by the fact that, under purely ballistic conditions (no optical phonon nor acoustic phonon scattering), the scattering mechanisms that cause the channel resistance to increase proportionally to channel length are neglected here.

**Figure 6:**
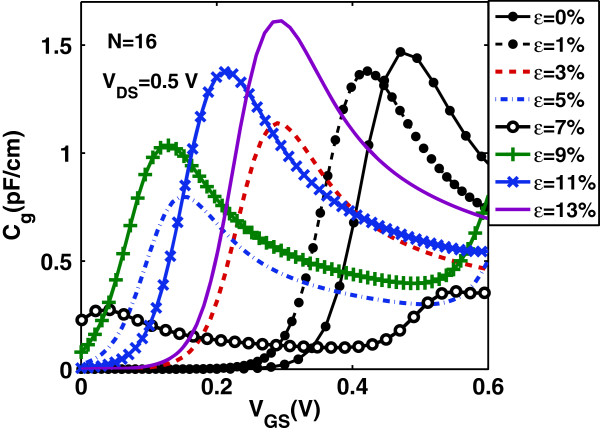
**Gate capacitance** ***C***_***g***_** versus** ***V***_**GS**_** for various tensile strains.**

**Figure 7:**
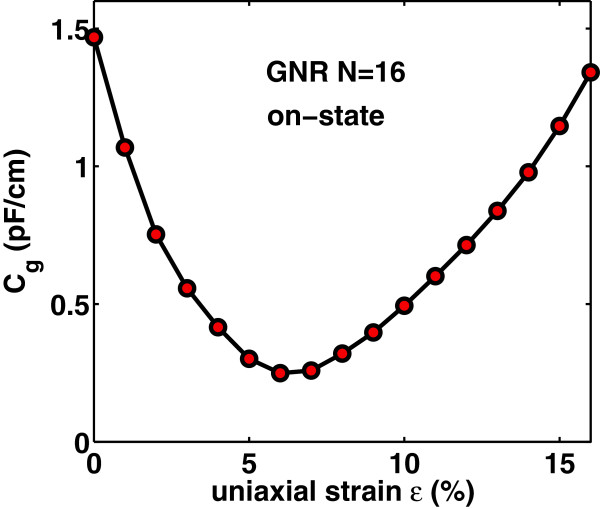
**Gate capacitance** ***C***_***g***_** versus uniaxial tensile strain in the ‘on-state’** ***V***_**GS**_**=** ***V***_**DS**_**=0** ***.*** **5 V.**

**Figure 8:**
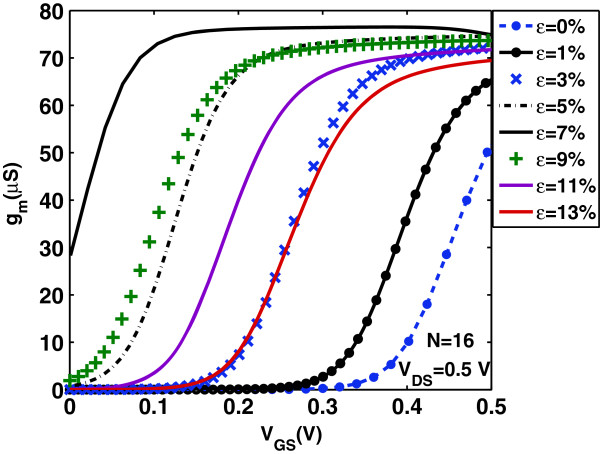
**Transconductance** ***g***_***m***_** versus** ***V***_**GS**_** for various tensile strains.**

**Figure 9:**
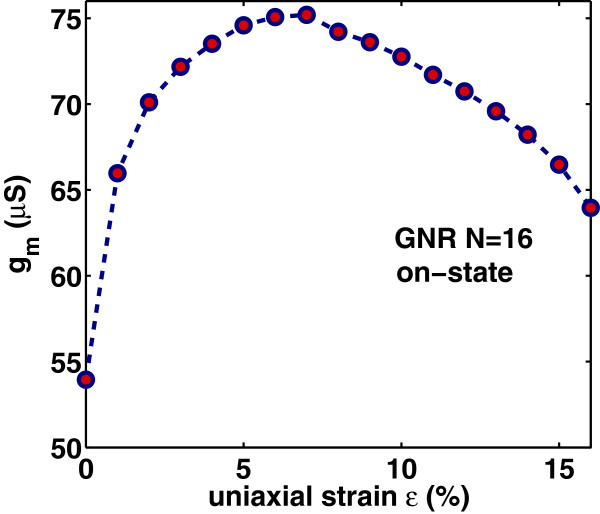
**Transconductance** ***g***_***m***_** versus uniaxial tensile strain in the ‘on-state’** ***V***_**GS**_**=** ***V***_**DS**_**=0** ***.*** **5 V.**

Now, we focus on the effect of uniaxial strain on the gate capacitance *C*_*g*_ and transconductance *g*_*m*_=*∂I*_D_/*∂V*_G_ of the device under study. Uniaxial strain changes the density of states and hence changes the quantum capacitance *C*_Q_ of the channel which is directly proportional to the density of states. As a result, in the quantum capacitance limit, uniaxial strain changes considerably the intrinsic gate capacitance *C*_*g*_. Figures 6 and 7 show *C*_*g*_ versus gate bias at drain bias *V*_DS_=0.5 V and *C*_*g*_ in the on-state (where *V*_GS_=*V*_DS_=*V*_DD_) versus strain *ε*, respectively. We clearly observe the non-monotonicity of the *C*_*g*_−*V*_G_ characteristics arising from the non-monotonic behavior of the function *F*_−3/2_(*x*) in Equation (11). A comparison of the curves in Figure 6 reveals that the gate bias *V*_G_ at which *C*_*g*_ peaks depends on the applied uniaxial strain. More specifically, the peak values of *C*_*g*_ are decreased and moved toward lower values of *V*_G_ as uniaxial strain is increased before the turning point and are increased and moved toward higher values of *V*_G_ as uniaxial strain is increased after the turning point. On the other hand, Figures 8 and 9 illustrate the effect of uniaxial strain on the transconductance *g*_*m*_ which describes the device’s switching-on behavior. As it is seen, *g*_*m*_ increases after threshold almost linearly with *V*_GS_ and does not peak at a certain gate voltage but gets saturated. Moreover, as uniaxial strain increases, *g*_*m*_ drastically increases from its value in the unstrained-GNR case, becomes maximum around the turning point *ε*≃7*%* and then decreases at a rate lower than that of the initial increase. This behavior follows the changes in carrier’s velocity with uniaxial strain, as explained earlier.

Next, we can assess the high-frequency performance potential of the device under strain. The cut-off frequency *f*_T_ is defined as the frequency at which the current gain becomes unity and indicates the maximum frequency at which signals can be propagated in the transistor. Once both gate capacitance and transconductance are calculated, *f*_T_ can be computed using the quasi-static approximation [38,39].

(15) $$f_{\text{T}}L_{\text{G}}=\frac{g_{m}}{2\pi C_{g}}{\left|\right)}_{V_{\text{D}}=V_{\text{DD}}}.$$

**Figure 10:**
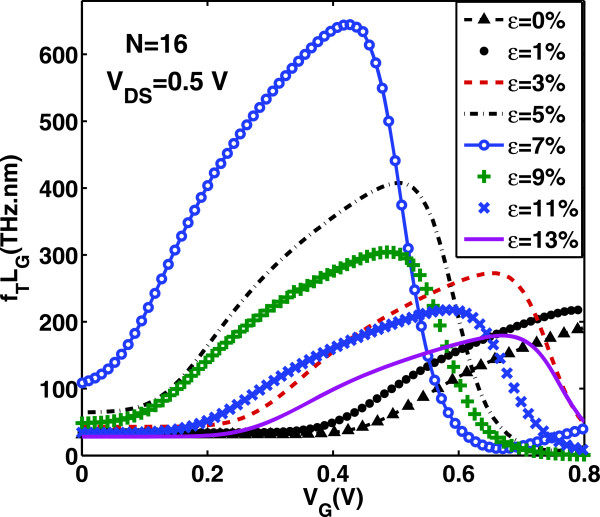
**Dependence of (*f***_**T**_***L***_**G**_**) on*V***_**GS**_** for various uniaxial strains.** The drain voltage is held constant at 0.5 V.

**Figure 11:**
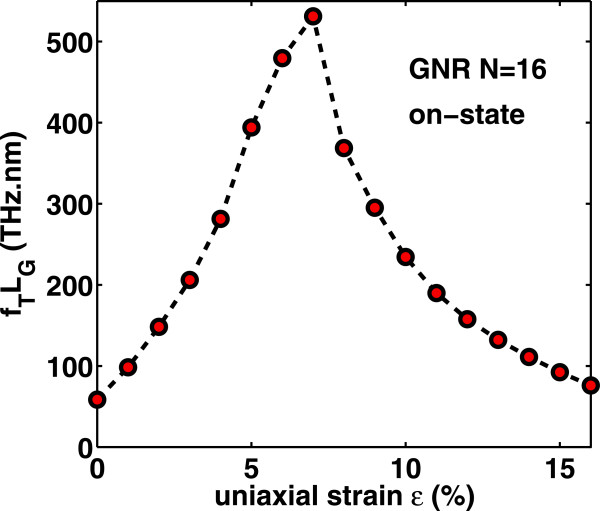
**Variation of (**$f_{\text{T}L_{\text{G}}}\;$**) with uniaxial tensile strain in the ‘on-state’** ***V***_**GS**_**=** ***V***_**DS**_**=0** ***.*** **5 V.**

It should be noted that a rigorous treatment beyond quasi-static approximation requires the inclusion of capacitive, resistive, and inductive elements in the calculation. In Figure 5, the quantity *f*_T_*L*_G_, where *L*_G_ is the channel length, as function of *V*_G_, for increasing values of uniaxial tensile stain, is depicted. Assuming a channel length of less than *L*_G_=50 nm, *f*_T_ exceeds the THz barrier throughout the bias window, confirming the excellent high-frequency potential of GNRs. Furthermore, Figures 10 and 11 show the variation of cutoff frequency versus gate voltage and strain *ε* (in the on-state), respectively. We clearly observe that *f*_T_ increases rapidly until the turning point *ε*≃7*%* and then decreases with lower rate for higher strain values (*ε*>7*%*). This is a direct consequence of both transconductance and gate capacitance variations with strain. Therefore, the high-frequency performance of AGNR-FETs improves with tensile uniaxial strain, before the ‘turning point’ of band gap variation but becomes worse after this point.

**Figure 12:**
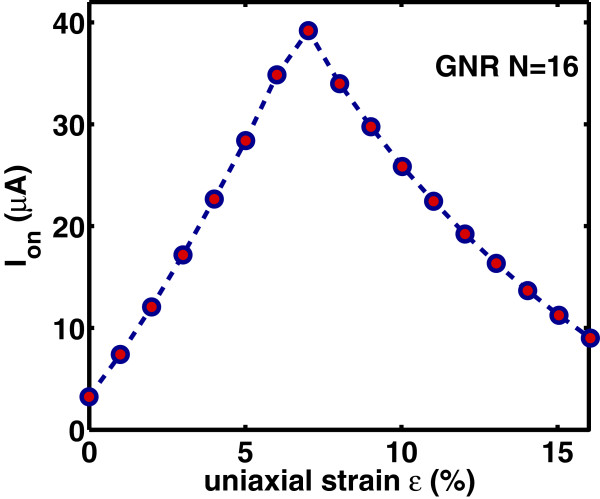
**Variation of the on-current** ***I***_**on**_** versus uniaxial strain.**

**Figure 13:**
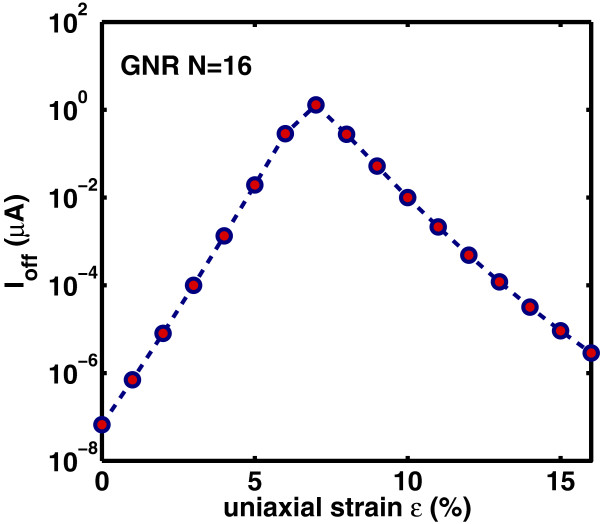
**Variation of the off-current** ***I***_**off**_** versus uniaxial strain.**

**Figure 14:**
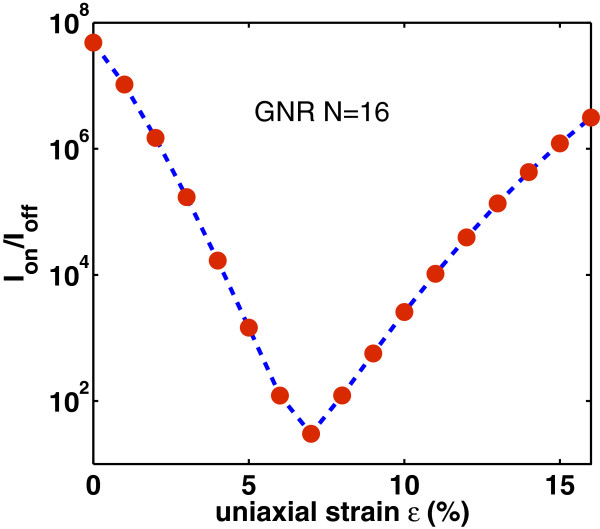
**Variation of the ratio** ***I***_**on**_**/*I***_**off**_** versus uniaxial strain.**

**Figure 15:**
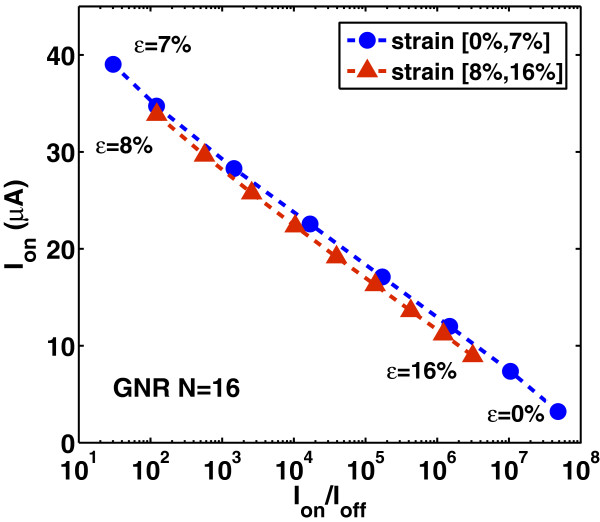
**Variation of** ***I***_**on**_** versus** ***I***_**on**_**/*I***_**off**_** ratio for various strain values.**

Lastly, we study the effect of strain on the switching performance of the DG-GNR FET. Figures 12, 13, and 14 show the dependence of *I*_on_, *I*_off_ and *I*_on_/*I*_off_ ratio on the uniaxial tensile strain, respectively. As it is clearly seen, the variation of both *I*_on_ and *I*_off_ is opposite to the variation of the band gap with strain whereas the ratio *I*_on_/*I*_off_ changes with strain following the band gap variation. The on-current *I*_on_ changes almost linearly with strain whereas the *I*_off_ and the ratio *I*_on_/*I*_off_ changes almost exponentially with strain. Note that the corresponding curves are not symmetric around the turning point, e.g., although for *ε*=12*%*, the GNR band gap returns to its unstrained value; the drain current at this stain value does not completely return to that of the unstrained GNR. This can be explained by the fact that although the band gap has returned its unstrained value, the carrier group velocity has been modified because, under tensile strain, some C-C bonds of the AGNR have been elongated [9]. Figure 15 shows the *I*_on_ versus *I*_on_/*I*_off_ plots for various strains which provides a useful guide for selecting device characteristics that can yield a desirable *I*_on_/*I*_off_ under strain. As it is seen, increased tensile strain before the turning point of band gap variation leads to lower *I*_on_/*I*_off_ ratio, whereas, increased tensile strain after the ‘turning point’ leads to higher *I*_on_/*I*_off_ ratio albeit at lower on-current.

**Figure 16:**
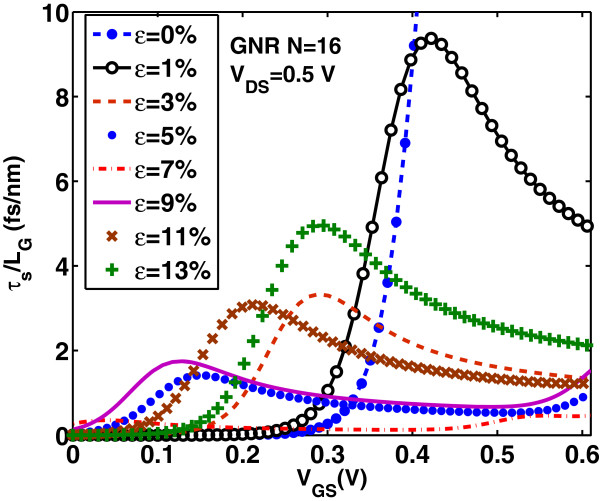
**Switching delay time** ***τ***_***s***_**/*L***_**G**_** versus gate voltage for various uniaxial strains.**

**Figure 17:**
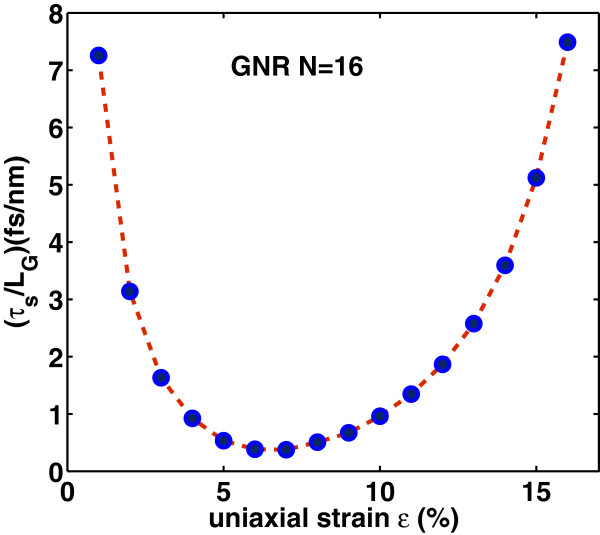
**Switching delay time*τ***_***s***_**/*L***_**G**_** versus uniaxial strain in the on-state*V***_**GS**_**=*V***_**DS**_**=0*.*5 V.** The delay time *τ*_*s*_/*L*_G_ for the unstrained case (*ε*=0*%*) (not shown) is found to be approximately 23 fs/nm.

**Figure 18:**
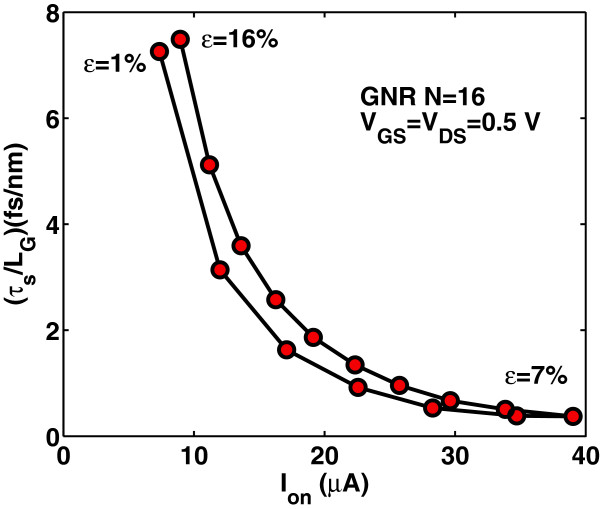
**Switching delay time** ***τ***_***s***_**/*L***_**G**_** versus on current** ***I***_**on**_** for various uniaxial strains.**

**Figure 19:**
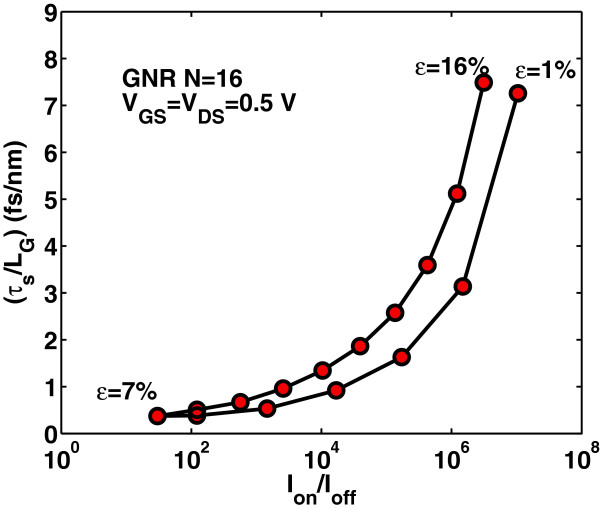
**Switching delay time** ***τ***_***s***_**/*L***_**G**_** versus** ***I***_**on**_**/*I***_**off**_**-ratio for various uniaxial strains.**

**Figure 20:**
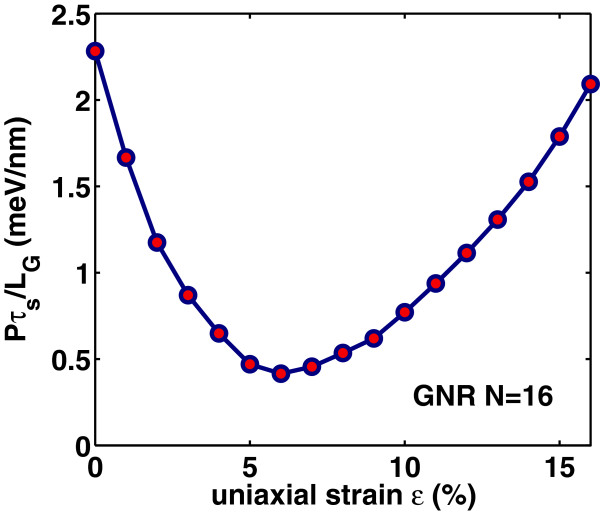
**Power-delay time product** ***P*** ***τ***_***s***_**/*L***_**G**_** versus uniaxial strain in the on-state** ***V***_**GS**_**=** ***V***_**DS**_**=0** ***.*** **5 V for various uniaxial strains.**

**Figure 21:**
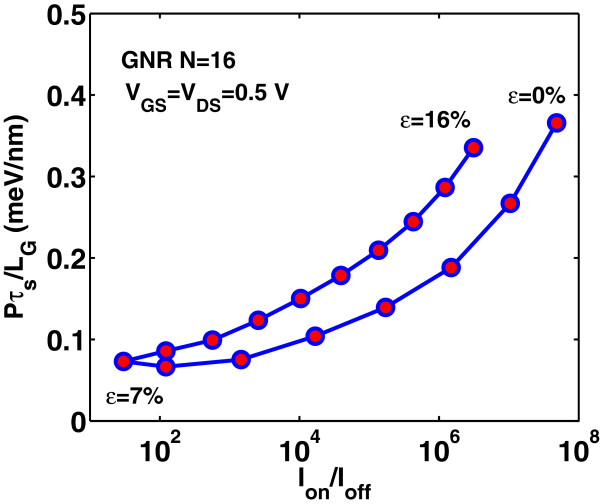
**Power-delay time product** ***P*** ***τ***_***s***_**/*L***_**G**_** versus** ***I***_**on**_**/*I***_**off**_**-ratio for various uniaxial strains.**

Intrinsic delay time *τ*_*s*_ is also an important performance metric that characterizes the limitations on switching speed and AC operation of a transistor. Once the gate capacitance is calculated, *τ*_*s*_ is given by [28].

(16) $$\tau_{s}=\frac{C_{g}V_{\text{DD}}}{I_{\text{on}}},$$

where the on-current is the drain current at *V*_G_= *V*_D_=*V*_DD_. Apparently, the switching delay time *τ*_*s*_ has similar variation as the gate capacitance has with strain, as it is depicted in Figure 16. Moreover, as it is seen from Figure 17, the switching delay time abruptly decreases with strain before the ‘turning point’ of band gap variation but increases rapidly after this point. We can say that switching performance improves with the tensile strain that results in smaller band gap whereas degrades with the tensile strain that results in a larger band gap. It is worth noting that the switching delay time for the unstrained case (*ε*=0*%*) is found to be *τ*_*s*_∼23 fs/nm, that is at least three times larger than the corresponding delay time in uniaxially strained-GNR case. Figures 18 and 19 show the switching delay time *τ*_*s*_ as a function of on-current *I*_on_ and *I*_on_/*I*_off_ ratio, respectively. For digital applications, high *I*_on_/*I*_off_ ratio and low switching time delay are required. However, when the *I*_on_/*I*_off_ ratio improves with the applied tensile strain, the *I*_on_ and switching performance degrade and vice versa. Another key parameter in the switching performance of the device is the power-delay product *Pτ*_*s*_=(*V*_DD_*I*_on_)*τ*_*s*_ that represents the energy consumed per switching event of the device. Figures 20 and 21 illustrate the dependence o of power-time delay product *Pτ*_*s*_ on strain and on *I*_on_/*I*_off_ ratio, respectively, where similar behavior to that of switching delay-time can be observed.

## Conclusions

We investigated the uniaxial tensile strain effects on the ultimate performance of a dual-gated AGNR FET, based on a fully analytical model. The model incorporates the effects of edge bond relaxation and third nearest neighbor (3NN) interaction as well as thermal broadening. We have focused on the AGNRs family *N*=3*p*+1 which is suitable for device applications. The strong modulation of I-V characteristics due to the changes in the strain is directly related to the electronic structure of the GNR channel region, which is modified as a result of changes in atomic structure under strain. The on-state current, gate capacitance, and intrinsic unity gain frequency are steadily improved for tensile strain less than the ‘turning point’ value of the band gap V-type variation. The observed trends are in consistency with the recently reported results based on tight-binding quantum transport numerical calculations [21-23]. Switching delay times improves with the tensile strain that results in smaller band gap whereas degrades with the tensile strain that results in a larger band gap. However, when the *I*_on_/*I*_off_ ratio improves with the applied tensile strain, the *I*_on_ and switching performance degrade and vice versa. Therefore, although a significant performance can be achieved by strain engineering, tradeoff issues should be carefully considered.

It is worthy noting that since purely ballistic transport and negligible parasitic capacitances are assumed, our calculations give an upper limit of the device performance metrics. Moreover, when metal-graphene contacts are used, the on-current of the ARGN-FET are degraded [40] by lowering the voltage drop on the intrinsic part of the device by a factor of *R*_bal_/(*R*_bal_+2*R*_cont_) where *R*_bal_ is the intrinsic resistance of the channel and *R*_cont_ is the contact resistances. Furthermore, in the presence of metal contacts, the cutoff frequency is degraded since the traversal time of carriers is significantly enhanced [41]. On the other hand, our approach may underestimate the actual concentration of carriers in the channel, especially for large drain and gate biases, when parabolic band misses to match the exact dispersion relation. However, we believe that the present fully analytical study provides an easy way for technology benchmarking and performance projection. Our study can be extended to compressive strain allowing negative values of uniaxial strain *ε* in our model. However, as it has been demonstrated [42], narrow GNRs exhibit a maximum asymmetry in tensile versus compressive strain induced mechanical instability, that is, the critical compressive strain for bucking is several orders of magnitude smaller than the critical tensile strain for fracture. Such a large asymmetry implies that strain engineering of GNR-devices is only viable with application of tensile strain but difficult with compressive strain.

## Competing interests

The author declares that he has no competing interests.
